# One-Port Electronic Detection Strategies for Improving Sensitivity in Piezoelectric Resonant Sensor Measurements

**DOI:** 10.3390/s16111781

**Published:** 2016-10-25

**Authors:** Zhongxu Hu, John Hedley, Neil Keegan, Julia Spoors, Barry Gallacher, Calum McNeil

**Affiliations:** 1 School of Mechanical and Systems Engineering, Newcastle University, Newcastle upon Tyne NE1 7RU, UK; zhongxu.hu@newcastle.ac.uk (Z.H.); barry.gallacher@newcastle.ac.uk (B.G.); 2 Institute of Cellular Medicine, Newcastle University, Newcastle upon Tyne NE1 7RU, UK; neil.keegan@newcastle.ac.uk (N.K.); julia.spoors@newcastle.ac.uk (J.S.); calum.mcneil@newcastle.ac.uk (C.M.)

**Keywords:** microsensors, piezoelectric resonators, self-sensing, frequency tracking

## Abstract

This paper describes a one-port mechanical resonance detection scheme utilized on a piezoelectric thin film driven silicon circular diaphragm resonator and discusses the limitations to such an approach in degenerate mode mass detection sensors. The sensor utilizes degenerated vibration modes of a radial symmetrical microstructure thereby providing both a sense and reference mode allowing for minimization of environmental effects on performance. The circular diaphragm resonator was fabricated with thickness of 4.5 µm and diameter of 140 µm. A PZT thin film of 0.75 µm was patterned on the top surface for the purposes of excitation and vibration sensing. The device showed a resonant frequency of 5.8 MHz for the (1, 1) mode. An electronic interface circuit was designed to cancel out the large static and parasitic capacitance allowing for electrical detection of the mechanical vibration thereby enabling the frequency split between the sense and reference mode to be measured accurately. The extracted motional current, proportional to the vibration velocity, was fed back to the drive to effectively increase the Q factor, and therefore device sensitivity, by more than a factor of 8. A software phase-locked loop was implemented to automatically track the resonant frequencies to allow for faster and accurate resonance detection. Results showed that by utilizing the absolute mode frequencies as an indication of sensor temperature, the variation in sensor temperature due to the heating from the drive electronics was accounted for and led to an ultimate measurement sensitivity of 2.3 Hz.

## 1. Introduction

Point-of-care biosensors will revolutionize our approach to healthcare and diagnostics. The promise of low cost, ease of use and wide range of analyte detection will see them applied to a range of targets. For a particular application, the target sensitivity is defined by the clinically relevant range for that analyte. From a generic sensor development viewpoint, the aim is therefore to maximize the achievable sensitivity thereby increasing the number of target markets to which the biosensor may be applied.

Along with the recent rapid advances in micro/nano fabrication technology, MEMS/NEMS resonator based sensors are becoming increasingly attractive in biosensing applications considering their major advantage of high sensitivity, the potential for miniaturization and integration, and low cost for large volume applications. They usually take the form of a cantilever [1], bridge [2], and similar structures that intend to have high quality factors. For sensors utilizing capacitive transduction, the electromechanical coupling factor is rather low and as the structure dimensions are scaled further down, mechanical thermal noise and preamplifier electronic thermal noise become dominant degrading the signal to noise ratio of the sensor output [3]. As a result, these devices require large DC bias, high amplitude of excitation, and need to be operated in a vacuum environment to recover the vibration signal [4]. Piezoelectric thin film devices have a much higher electromechanical coupling coefficient than electrostatic capacitive transduction due to difficulty in gap fabrication for capacitive based sensors [5]. This makes it a promising replacement of electrostatics in device drive and vibration sensing in resonant sensors using its direct and converse piezoelectric effects [6,7]. A range of reported flexural piezoelectric biosensor designs are given in Appendix A.1.

In the development of piezoelectric devices, measurements are typically characterized either optically [8,9,10,11] or by using an impedance analyzer [10,12,13,14,15,16], alternatives include a DSP signal processed voltage variation from a sense electrode [9] and a modified Pierce oscillating circuit used to send the cantilever into self-oscillation [12]. Commercially, MEMS resonant sensors are controlled by different forms of phase-locked loop or self-sustained oscillator circuits for automatically tracking the resonance frequency shift as a measurement output, for example, the control system for the quartz crystal microbalance (QCM) [17]. The sensitivity and resolution of such sensors are determined by the phase noise or the short term frequency stability. The phase stability of such sensor systems is fundamentally limited by the noise associated with the MEMS resonator’s mechanical thermal noise and the electrical thermal noise of the piezoelectric material. Practically, the phase stability is often dominated by the electronics noise from the pre-amplifier, flicker noise, and noise of the power supply. Self-heating and temperature fluctuation also significantly affect the frequency stability for piezoelectric driven devices. The stability of the resonant frequency and the accuracy to which it can be measured ultimately decide the sensitivity, and thus the range, of clinically relevant targets the sensor can be realistically applied to.

Two general configurations exist for electrically driving and sensing piezoelectric devices. The two port configuration uses separate piezoelectric electrodes for drive and sensing whereas the one-port configuration may be classed as self-sensing as it utilizes the drive current to monitor device response. The latter has the advantage of simpler interface electronics. One-port examples include self-oscillating circuits using a MEMS device as part of an oscillator circuit [18], impedance based modal analysis [19], structural damping and vibration control using a single PZT (lead zirconate titanate) element [20] and quartz micro balance [17]. Capacitive bridge and other compensation techniques have been developed to mitigate for parasitic capacitance influences particularly in one-port sensor applications [20]. However, temperature influence on piezoelectric strain constant d_31_ and permeability e_33_ affects performance of such circuits [21]. Simmers et al. [22] propose that adding a capacitor in series or parallel with both the PZT and the matching capacitor can improve temperature stability. This is based on the idea that adding capacitance to the circuit would produce a smaller change in the capacitance mismatch between parasitic and matching capacitances in the bridge circuit.

The circular diaphragm resonant (CDR) biomass sensor [23] is targeted at point-of-care applications and is based around the idea of utilizing degenerate resonant modes of vibration that offers insensitivity to environmental fluctuations due to its differential measurement scheme. For the CDR biosensor being assessed in this work, the direct improvements from utilizing an integrated piezoelectric thin film in this sensor are a simplified fabrication process, low motional resistance and high motional capacitance leading to improved signal to noise ratio, reduced cross talk, and simplified signal recovery as compared to the capacitively-driven/sensed version [4,24]. A comparison between piezoelectric and capacitively-based versions is given in Appendix A.2. The choice of PZT over other piezoelectric material choices was due to its large piezoelectric coefficients. However due to the loss in crystallinity in this composite structure, quality factors Q are typically reduced compared to pure silicon resonators.

The aim of this work is to assess approaches to electronic recovery of signal from a piezoelectric resonator and assess frequency tracking performance and sensitivity from such an approach. This paper looks to implement a one-port positive current feedback scheme. Firstly the equivalent electrical circuit model of the piezoelectric thin film driven resonator is examined and based on this, parasitic compensation is implemented. With parasitic current effectively cancelled, pure motional current may be extracted allowing for current feedback to the drive to improve the Q factor. Finally, the work looks to implement automatic resonant frequency tracking by a phase-locked loop. The work looks to validate the approach by implementation of the technique on a microfabricated circular diaphragm resonant sensor and assess the sensitivity from such an approach.

## 2. Design and Interface Considerations

To implement the sensing and control electronics, an equivalent electrical circuit model for the mechanical resonant sensor is derived. The model contains a large parasitic capacitance term originating from the relative proximity of the signal tracks to one another. A compensation circuit is utilized to remove this parasitic capacitance leading to an output signal representative of the motion of the resonator. The output signal is then fed back to drive the device resulting in a bandwidth reduction of the resonating mode. Finally an all-digital phase-locked loop frequency tracking scheme is implemented around a programmable lock-in amplifier to allow for tracking of the frequencies of the sensing modes of the device. Before detailing the electronics development, an overview of the sensor design is given.

### 2.1. Sensor Design

The cyclically symmetric circular diaphragm resonator supports pairs of independent modes of vibration which share a common natural frequency referred to as degenerate modes. These modes are defined by the nodal diameter and nodal circle numbers (n, m) [25], where in this design it is the (1, 1) mode that is utilized for sensing. Immobilization of mass over predefined regions on the diaphragm preferentially adds modal mass to one of these modes causing a breaking of the degeneracy and producing a frequency split proportional to the added mass [23]. For a perfectly fabricated (symmetric) structure there would initially be—i.e., prior to mass addition—a zero frequency split between degenerate modes. Due to the symmetry of the design, ambient effects such as temperature and isotropic in-plane stress equally influence both modes and therefore do not affect the frequency split.

**Figure 1:**
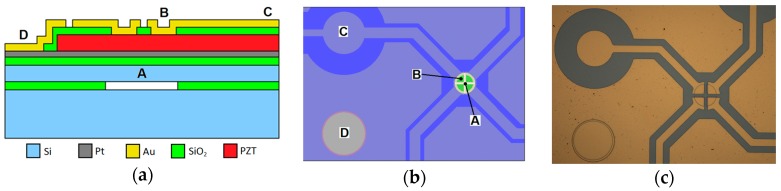
(**a**) Schematic cross section of the device (not to scale). ‘A’ indicates the resonator, ‘B’ is the top electrode contact, ‘C’ is the top electrode bond pad, ‘D’ is the ground plane electrode contact; (**b**) Screenshot of mask indicating lateral position of the features; (**c**) A microscope image of a fabricated device.

Fabrication of the sensors involved the fusion bonding of two silicon wafers to form a 4.5 µm thick, 140 µm diameter diaphragm suspended over a sealed cavity. Surface micromachining was then used to create 750 nm thick PZT drive/sense regions on the sensor surface with a common platinum ground electrode and separate gold electrodes on the PZT upper surface. A schematic cross-section of the sensor is shown in Figure 1a. In-plane electrode arrangement and a microscope image of a fabricated device is shown in Figure 1b,c respectively. A 3D exploded view of the sensor is given in Appendix A.3. Full details on design and fabrication may be found in Hu et al. [26].

### 2.2. Electrical Equivalent Circuit Model

**Figure 2:**
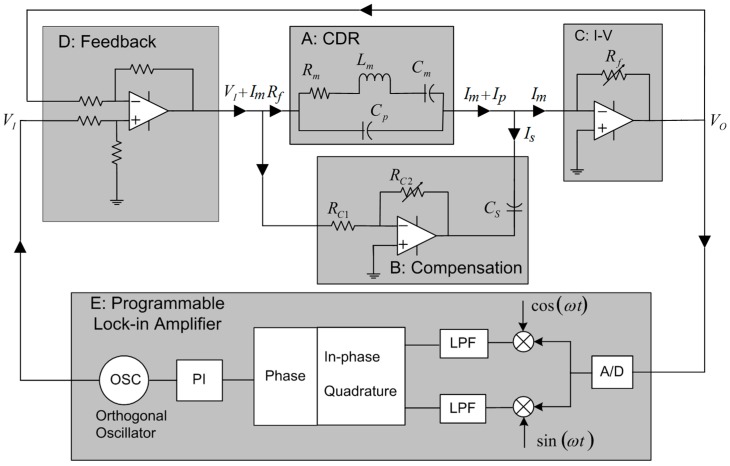
(**Sub-circuit A**): The equivalent circuit model of the CDR; (**Sub-circuit B**): Circuit used to compensate for the parasitic capacitance present; (**Sub-circuit C**): Current to voltage converter, nominal value of *R_f_* is 100 Ω; (**Sub-circuit D**): A current feedback scheme is used to increase the Q of the system; (**Sub-circuit E**): A digital lock-in amplifier arrangement (Zurich HF2) is used to track the resonant frequency.

While designing the interface electronics to a resonator sensor, it is convenient to model the mechanical resonator by an electrical equivalent circuit consisting of motional inductance, capacitance, and resistance in parallel with a parasitic capacitance [19,27]. The equivalent electrical circuit used to model the dynamical behaviour of the resonator and electromechanical coupling between the PZT thin films with the resonator is shown in Figure 2 sub-circuit A, further details given in Appendix A.4. The motional inductance *L_m_* is related to the oscillating mass, motional capacitance *C_m_* is related to material elasticity, and motional resistance *R_m_* represents the dissipation effect from friction and acoustic damping. Motional resistance is a critical parameter in the design of sensor systems including self-oscillation circuits, phase-locked loop based resonant frequency tracking, or simply frequency sweeping [28]. *C_p_* is the parasitic capacitance which is relatively large when compared with electrostatic or crystal resonators [29].

The dynamic behaviour of the CDR resonator can be characterized by examining the impedance (or admittance) function versus frequency of the equivalent circuit, given by:

(1) $$Z\left(j\omega \right)=\frac{\left(1-\omega^{2}L_{m}C_{m}\right)+j\omega R_{m}C_{m}}{-\omega^{2}R_{m}C_{m}C_{p}+j\omega \left(C_{m}+C_{p}-\omega^{2}L_{m}C_{m}C_{p}\right)}$$

The frequency response of Equation (1) shows a local minimum and maximum respectively corresponding to series frequency *ω_s_* and parallel frequency *ω_p_* which are defined as:

(2) $$\omega_{s}=\frac{1}{\sqrt{L_{m}C_{m}}}$$

(3) $$\omega_{p}=\omega_{s}\sqrt{1+\frac{C_{m}}{C_{p}}}$$

It can be seen from Equation (3) that the parallel frequency *ω_p_* is always greater than *ω_s_*. Large parasitic capacitance causes the series and parallel frequencies to be very close to each other, making it difficult to accurately detect the resonant frequency. In particular, the distorted phase response prevents self-oscillation which relies on zero phase at resonance. Knowing the motional resistance *R_m_*, the quality factor of the series resonance frequency Q is determined by equation:

(4) $$Q=\frac{\omega_{s}L_{m}}{R_{m}}$$

For an accurate measurement of the Q factor from a simple frequency response, the static capacitance must be compensated.

In most of the interface circuit designs, it is more convenient to use admittance of the electrical equivalent circuit, as it is linearly related to the current flow through the device:

(5) $$Y\left(j\omega \right)=\frac{\omega^{2}R_{m}{C_{m}}^{2}}{{\left(1-\omega^{2}L_{m}C_{m}\right)}^{2}+\omega^{2}{R_{m}}^{2}{C_{m}}^{2}}+\frac{j\omega C_{m}\left(1-\omega^{2}L_{m}C_{m}\right)}{{\left(1-\omega^{2}L_{m}C_{m}\right)}^{2}+\omega^{2}{R_{m}}^{2}{C_{m}}^{2}}+j\omega C_{p}$$

The phase response of the admittance at series resonant frequency is given by:

(6) $$\varphi \left(j\omega_{s}\right)=\tan^{-1}\left(\omega_{s}R_{m}\left(C_{m}+C_{p}\right)\right)=\tan^{-1}\left(\frac{\left(C_{m}+C_{p}\right)}{QC_{m}}\right)$$

### 2.3. Parasitic Compensation

Often, the impedance of the parallel parasitic capacitance is significant when compared to the motional resistance presenting a major challenge in a sensor system design to achieve high signal to noise ratio. From Equation (6), it is evident that the parasitic capacitance *C_p_* shifts the phase angle at resonance and will cause a frequency error for sensor systems that are based on algorithms of zero phase tracking at resonance. Due to the inherently large dielectric coefficient of PZT, the influence of parasitic capacitance on the phase and amplitude is significant in this work. It is therefore vital to eliminate the influence of this parasitic capacitance *C_p_*.

There are several static capacitance compensation circuits reported in literature, for example, in piezoelectric vibration control [20] and quartz crystal micro balance applications [30,31]. The compensation technique used in this work is implemented through sub-circuit B shown in Figure 2. Current flowing through parasitic capacitance *C_p_*, plus any additional parasitic capacitance added by the control circuitry, is cancelled by current passing through *C_s_*, tuned by finely trimming feedback potentiometer *R_C_*_2_ of the inverting amplifier. The transfer function, in terms of transform variable frequency parameter s, from input drive signal *V_I_* to output signal *V_O_* is described as:

(7) $$\frac{V_{o}\left(s\right)}{V_{I}\left(s\right)}=-R_{f}\left[\frac{1}{R_{m}+sL_{m}+\frac{1}{sC_{m}}}+s\left(C_{p}-\eta C_{s}\right)\right]$$

With $\eta =R_{C2}/R_{C1}$ and *R_f_* the feedback resistance for current-to-voltage conversion, shown as sub-circuit C in Figure 2. It is clear from the transfer function, Equation (7), that by adjusting compensation gain *η* using potentiometer *R_C_*_2_, the parasitic capacitance *C_p_* role can be cancelled from the transfer function by the compensating capacitance *C_s_*. At 100% compensation, the frequency response becomes solely the admittance of the resonator without parasitic effect.

### 2.4. Motional Current Feedback

A high quality factor is a major parameter for resonance sensors to achieve high sensitivity. One particular technique which has been widely reported is Q amplification, particularly in AFM [32] and similar applications [33]. In these, optical sensors are used to detect displacement of a resonant beam and the velocity of vibration extracted from phase shifting the displacement signal and feeding it back to the drive. For a one-port sensor configuration, using a single PZT thin film as both drive and sensing, the velocity can be estimated from the motional current.

The output signal from the interface sub-circuit, *V_o_* in Figure 2, is a measure of all the three currents, motional current *I_m_*, parasitic current *I_p_*, and compensation current *I_s_*. When *I_p_* and *I_s_* cancel each other, the output signal reflects solely the motional current. By feeding the output voltage back to the drive, as shown in sub-circuit D of Figure 2, the Q factor of the resonance can be improved. This feedback control scheme is similar to velocity feedback algorithms seen [32,33] however here we provide a form for the one-port sensor configuration with significantly simplified implementation. As no analog filters or differentiators—which usually introduce electronic noise and only work within a narrow frequency range around the resonance—are required with this approach, this direct current feedback scheme has the additional advantage of a high signal to noise ratio.

The transfer function of the sensor system with current feedback is described as:

(8) $$\frac{V_{o}\left(s\right)}{V_{I}\left(s\right)}=\frac{\left(S^{2}L_{m}C_{m}+SR_{m}C_{m}+1\right)SR_{f}\left(C_{p}-\eta C_{s}\right)-SR_{f}C_{m}}{S^{2}L_{m}C_{m}+S\left(R_{m}-R_{f}\right)C_{m}+1+\left(S^{2}L_{m}C_{m}+SR_{m}C_{m}+1\right)SR_{f}\left(C_{p}-\eta C_{s}\right)}$$

When the parasitic capacitance *C_p_* is completely cancelled by adjusting *R_C_*_2_, that is $C_{p}=\eta C_{s}$, the transfer function is simplified as:

(9) $$\frac{V_{o}\left(s\right)}{V_{I}\left(s\right)}=-\frac{SR_{f}C_{m}}{S^{2}L_{m}C_{m}+S\left(R_{m}-R_{f}\right)C_{m}+1}$$

From Equation (9) it is clear the dissipation can be reduced by feedback control, which is controlled by resistor *R_f_*. The Q factor:

(10) $$Q=\frac{\omega_{s}L_{m}}{R_{m}-R_{f}}$$

becomes infinite when *R_f_* equals the motional resistance *R_m_* of the device. Further increasing *R_f_* can lead to instability due to negative damping. Practically, *R_f_* is set to a lower value to avoid self-oscillation of the amplifier due to electronic noise.

### 2.5. Phase-Locked Loop Resonance Tracking

Resonance sensors can be designed to operate in different modes. Open loop techniques include frequency sweep to reveal resonance frequency shift, and measuring phase shift or variation of amplitude by driving the resonator with fixed frequency signal around the resonance. Changes in the resonance frequency, phase shift, or amplitude of response are then monitored. A commonly used closed loop detection scheme is a phase-locked loop that automatically locks the drive signal at either zero or 90° depending on whether velocity or displacement is sensed. Closed loop detection is faster and can have higher frequency resolution by increasing control gains.

After static capacitance is compensated, the motional current is left as the measurement output which is proportional to the velocity of vibration. A digitally-implemented phase-locked loop was used to lock the drive frequency at 0°. The system diagram is described in sub-circuit E of Figure 2.

## 3. Experimental Details

**Figure 3:**
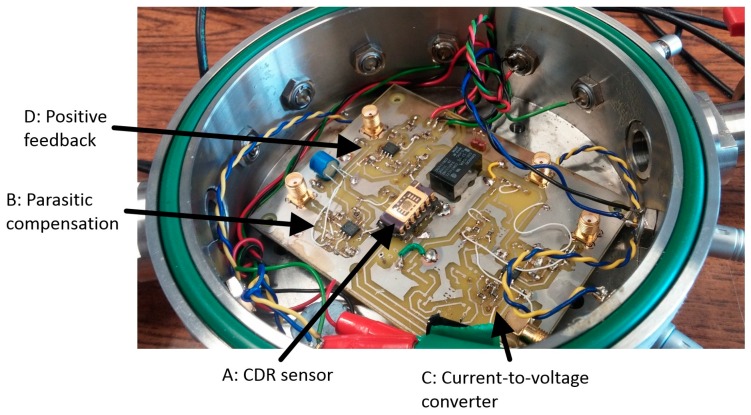
The CDR sensor and control circuitry.

**Figure 4:**
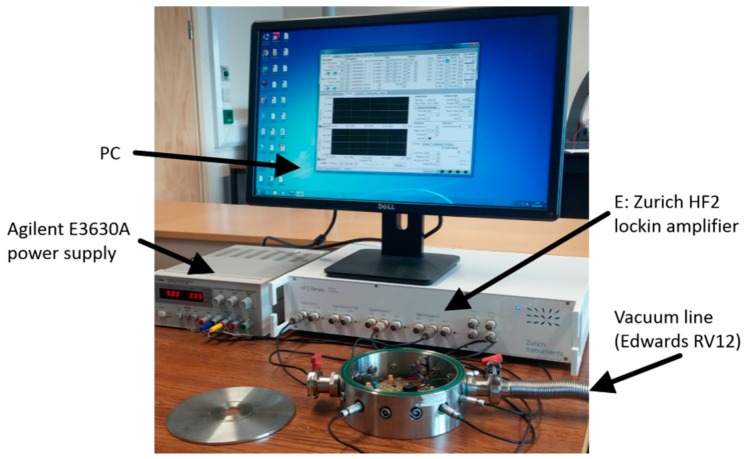
The experimental setup used to test the CDR sensor.

Images of the circuit and experimental setup are shown in Figure 3 and Figure 4 respectively with labelling A to E representing the sub-circuits as described in Figure 2.

Measurements were predominantly recorded at atmospheric pressure. As variations in air flow over the device affects the device and electronic components, the final assessment of tracking performance was conducted under vacuum conditions. For those measurements taken in a vacuum, the device and corresponding circuitry was placed in a vacuum chamber evacuated to <1 mbar using an Edwards RV12 vacuum pump. The sub-circuits were constructed using ultra-low noise op amps LMH6626 with typical input voltage noise of 0.92 nV/√Hz and a bandwidth of 1.5 GHz. A nominal resistance of 100 Ω was used to convert current to voltage and increased when assessing the current feedback scheme.

Devices were driven with a 10 mV drive signal during parasitic capacitance compensation and with a 1 mV drive when feedback was implemented. Higher drive voltages would result in non-linear resonant behaviour of the device. Data was recorded via a Zurich HF2 lock-in amplifier. The lock-in was programmed using MATLAB to calculate amplitude and phase and lock the phase to the required set point. Phase shifts introduced by the circuit were compensated for in software. Data was sampled at 240 MHz and 14 bit resolution, digitally demodulated, and the resulting amplitude and phase information saved to PC.

For resonance tracking, orthogonal demodulation was carried out within the lock-in amplifier to calculate the in-phase and quadrature components relative to the drive. The calculated phase error was then used to set an appropriate frequency correction for the drive by PI control algorithms that close the control loop. The software phase-locked loop tracked the resonance frequencies of the degenerated modes pairs, denoted as mode 1 and mode 2, in an interleaved pattern so not to cause mode interaction.

## 4. Results and Discussion

In this section, the performance of the parasitic capacitance compensation and current feedback will be assessed. Self-heating and temperature fluctuation are found to be the major causes of frequency instability because of the high active loss and low heat capacity of the PZT thin film. Despite the detrimental temperature drift on measurement resolution, a novel solution to address temperature variation is proposed that improves the frequency resolution of the devices.

### 4.1. Parasitic Compensation

**Figure 5:**
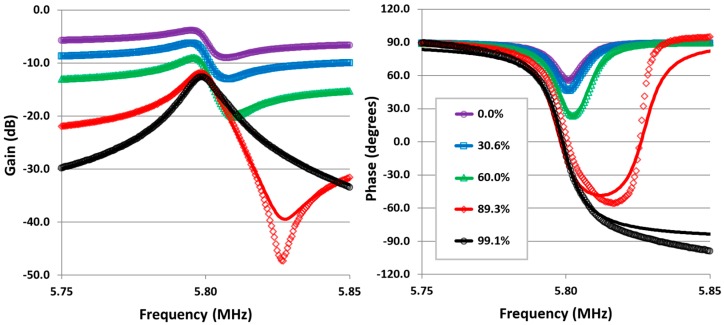
Amplitude and phase response traces for a CDR device. Legend refers to how much parasitic capacitance has been compensated for (100% corresponds to fully compensated). Points represent experimental data, solid lines represent a least squares fitted model—described by Equation (7)—to the experimental data.

Figure 5 shows the results of a device tested with the parasitic compensation circuit, the level of compensation being defined as $1-\left(C_{p}-\eta C_{s}\right)/C_{p}$. The tests were conducted at atmospheric pressure with drive signal amplitude of 10 mV, feedback resistor *R_f_* = 100 Ω. The results demonstrate piezoelectric thin film based CDR devices only need a drive amplitude of millivolts to give an acceptable signal response—i.e., signal to noise ratio in excess of 10—when operated in air. The major parameters *L_m_*, *C_m_* of a fabricated device can be estimated from a fitting of the experimentally obtained resonance curve, either amplitude or phase, to the expected transfer function given in Equation (7). Table 1 shows the major parameters of the CDR device tested, the results are an average of the fitting parameters (for both amplitude and phase plots) for the five levels of compensation tested. This gives values for series and parallel frequencies of 5.790230 MHz and 5.793338 MHz respectively and a Q factor of 507.

Note the phase response swings from +90° to −90° for perfect compensation which is vital for automatic resonance frequency tracking control. As the mode shape will inevitably be misaligned to some degree with respect to the drive electrodes due to manufacturing tolerances, the discrepancy between theoretical and actual responses, particularly in the phase plot, is attributed to a contributing signal from the second resonance of the mode pair [26].

### 4.2. Motional Current Feedback

**Figure 6:**
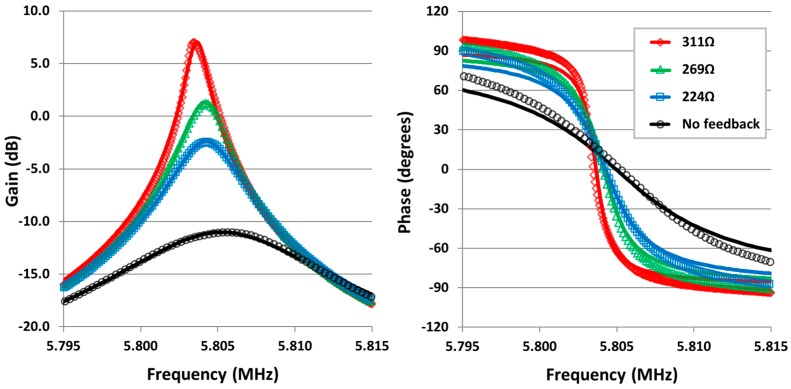
Frequency responses of piezoelectric based CDR with current feedback. Points represent experimental data, solid lines represent a least squares fitted model—described by Equation (9)—to the experimental data. Legend indicates values of feedback resistor *R_f_*.

For current feedback Q amplification testing, the feedback resistor *R_f_* was adjusted. Results for a tested device are shown in Figure 6 and quantified in Table 2. The feedback gives a sharper resonant peak with eight-times amplification of Q at the largest feedback resistance used, greater rate of change of phase through resonance and therefore a more precise measurement of the resonance frequency. There is a slight frequency shift along with increased feedback level, this is caused by the small phase shift at the output signal due to limited gain bandwidth product of the amplifier [34]. The results show frequency shifts up to −1.5 kHz. Phase compensation of the current feedback signal could be implemented to eliminate this shift however, due to the differential measurement principle of the CDR, this small frequency shift—seen in both modes—is automatically compensated for.

### 4.3. Phase-Locked Loop Resonance Tracking

**Figure 7:**
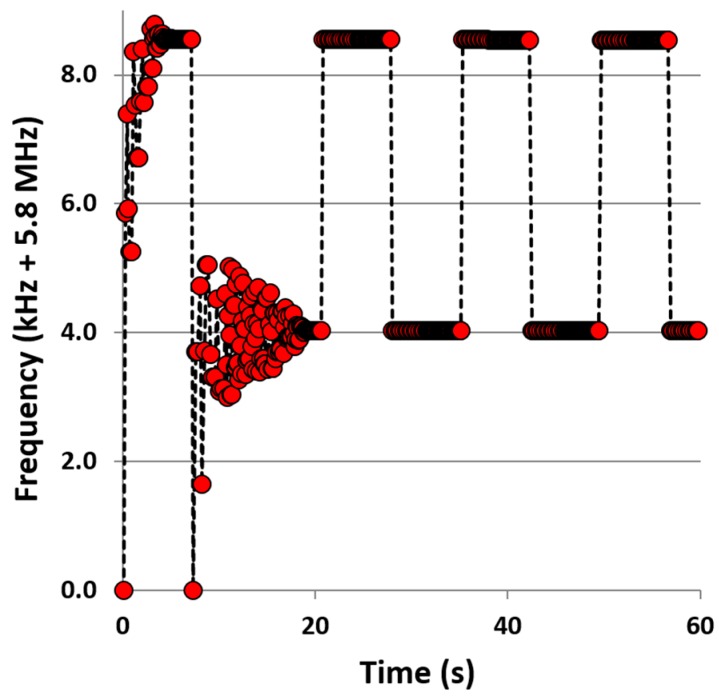
Frequency tracking is performed by phase-locking each mode. A mode is locked, the frequency is recorded and then the tracking system moves onto locking the other mode. This process continues to allow for assessing the frequency drift of each mode and allows for calculation of the frequency split. Once each mode is initially locked, relocating and locking takes approximately 7 s. The initial starting frequency is 5.8 MHz.

To assess the sensitivity achievable from this approach, a device was tested under vacuum conditions with drive signal amplitude of 1 mV. With proportional and integral gains of 5 and 1 respectively, the lock-in amplifier was programmed to lock onto each of the (1, 1) degenerate resonance modes, record that frequency and then switch to the other mode. Phase noise was of the order of 0.01° and it was therefore decided to lock phase to with 0.05°. Time of lock, resonant frequency and phase were recorded. Figure 7 shows the lock-in performance of the system. The system took around 7 s to lock onto each resonant frequency, this time being predominantly the required period for the feedback scenario to ring up the oscillation. The system’s electronic noise was quantified by assessment of the rms of the frequency scatter of the recorded data during phase-locking. A second order polynomial was fit to the data to account for frequency drift due to temperature variations and the remaining rms scatter was measured at 0.3 ± 0.1 Hz.

**Figure 8:**
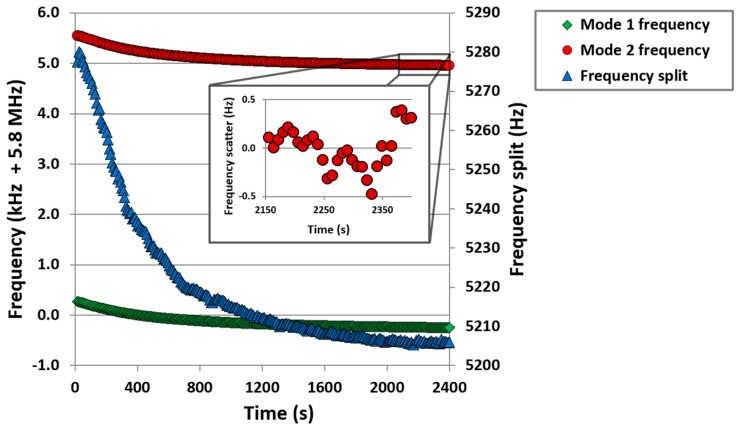
Frequency tracking tests of resonance frequency shifts induced by environmental temperature variations (green and red lines indicate resonance frequencies of modes 1 and 2 respectively, blue trace is frequency split between these modes). The scatter from a linear fit of mode 2 from 2150 s (inset) demonstrates resolution of frequency tracking is within 0.5 Hz.

The current feedback leads to a steeper phase gradient with a phase sensitivity of 0.086°/Hz at resonance. For the 0.05° phase-lock, this corresponds to an ultimate frequency tracking performance of 0.58 Hz. Although tracking performance of each mode reflects this performance, as shown in Figure 8, a 40 min tracking of the frequency split showed a variation of 75 Hz, the frequency split being calculated from the mode 1 resonance frequency minus an interpolated mode 2 value so time of measurement is coincident.

**Figure 9:**
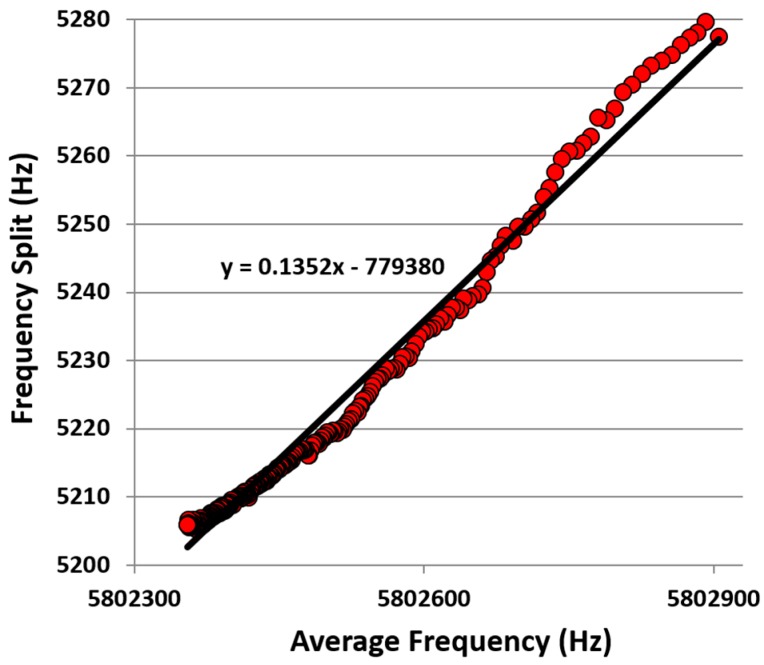
Frequency tracking tests of resonance frequency shifts induced by environmental temperature variations. Although frequency split should theoretically be temperature invariant, manufacturing tolerances result in a slight temperature sensitivity. As average frequency is highly indicative of temperature, this may be used to help compensate for temperature induced variations in frequency split.

Although in theory the frequency split measurement of the CDR should be insensitive to temperature, manufacturing tolerances lead to some temperature sensitivity [26,35]. With these piezoelectric CDR sensors showing a resonant frequency temperature sensitivity of typically −75 ppm·°C^−1^ [26], the change in absolute frequency shown in Figure 8 corresponds to a temperature drift of only 1–2 °C for the duration of the measurement. Experimentally, temperature could be tightly controlled to obtain optimum performance from the device however this becomes impractical in point-of-care scenarios. However, as the resonant frequencies have high temperature sensitivity, they can therefore be used to correct for temperature variations. The current used to drive the device is expected to locally heat the device, causing a slight expansion with respect to the cooler silicon support die, thereby introducing compressive stress within the device and a lowering of the resonant frequency. A re-plotting of Figure 8 of average frequency of the two modes against frequency split, see Figure 9, indicates the achievable consistency in the measurement as temperature varies. Variation of data points to the best fit line shows an rms value of 2.3 Hz which, for these devices, correspond to a modelled mass resolution of 160 fg.

### 4.4. Implications to Mass Measurement Protocols

**Figure 10:**
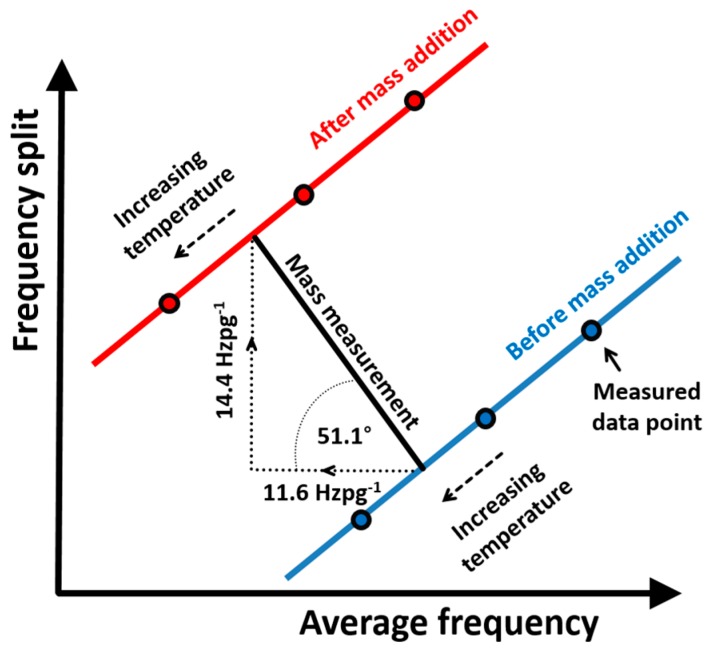
A range of measurements of average frequency and frequency split before and after mass addition may be used to compensate for temperature variations.

Mass addition onto the sensor would lead to a drop in average resonant frequency and an increase in frequency split. This would have the effect of moving the best fit line shown in Figure 9 to the left and upwards, respectively. The target analyte to be measured would, in practice, be introduced to the sensor through a microfluidic setup. The unspecified temperature of this analyte would inevitably lead to a temperature change in the sensor thereby making it impossible for an accurate determination of added mass from a single frequency split measurement. For accurate measurements, the sensor’s temperature sensitivity needs to be accounted for and the heating effect from the actuation provides a convenient opportunity to provide this information. Figure 10 demonstrates the measurement scenario. Finite element modelling indicates that a picogram of mass selectively added to the sensor surface would induce a change in frequency split of 14.4 Hz whilst lowering the average frequency of the two modes by 11.6 Hz. Several resonant frequency measurements before and after mass addition at slightly varying temperatures allows for determination of the displacement of the best fit lines to the frequency tracking measurements thereby inferring the frequency change at a constant temperature and thus a more accurate measurement of the quantity of added mass.

### 4.5. Comparative Performance of the Device

The detection scheme proposed in this work has demonstrated a frequency resolution of 2.3 Hz in the 5.8 MHz resonators corresponding to a measurement limit of 0.4 ppm. This compares favourably with the 13 ppm previously reported for the piezoelectric CDR [26] and for other flexural piezoelectric biosensors [8,9,13,14,15] with demonstrated lower mass detection limits corresponding to measurement limits of 1000–6000 ppm. In setting the feedback resistance, R_f_, the motional resistance, R_m_, was determined with no feedback, R_f_ set lower than this and then incrementally increased until the resonance appeared non-linear (i.e., not symmetric). This gave a factor of 8 improvement in Q leading to a shaper phase response, better frequency tracking and ultimately higher mass sensitivity. In practice, the drive signal amplitude may be further reduced allowing for larger values for R_f_ giving further improvements in Q and mass sensitivity. Realistically, Q amplification is limited by the non-negligible homogeneous term in the system response, namely the difference term between R_m_ and R_f_, as Q becomes very sensitive to the ability to set R_f_ as R_f_ approaches R_m_. High Q amplification is also sensitive to the noise characteristics of the electronic circuit and so further enhancements in Q would require using electronic circuitry with superior noise characteristics.

Temperature controlled QCM [36,37,38] still demonstrates superior performance at 0.01 ppm measurement limit however stringent temperature control surpassing ±0.03 °C is required to achieve this level which becomes impractical for point-of-care scenarios. Although the proposed solution in this work allows for automatic correction of temperature variation within the device, detection electronics must still be protected from temperature fluctuations to achieve the 0.4 ppm performance reported.

## 5. Conclusions

This work presented interface electronics for resonance detection and control for a piezoelectric thin film driven silicon circular diaphragm resonator designed for use as a biomass sensor. In comparison to capacitive designs, the major advantage of using PZT thin films in devices as a means of vibration drive and sensing are significantly improved Q factor in air and simplified fabrication process, electronics interface, and control.

A common characteristic of piezoelectric thin film based resonant devices is the large static and parasitic capacitance that leads to the co-existence of series and parallel resonances. This causes reduced frequency resolution and prevents automatic frequency tracking control. A compensation circuit was designed to minimize the parasitic influences and allow for more accurate detection of resonance frequency. This compensation scheme also allows for the mechanical vibration related motional current to be extracted and fed back to the drive, which has effectively improved the Q factor from around 500 in air by more than a factor of 8. The scheme did introduce a frequency shift in the measurement of the resonance peak of 0.03% for this amplification which is automatically compensated in the frequency split measurement of this device. Automatic resonance tracking using a phase-locked loop was implemented that showed fast and accurate detection of the resonance frequencies.

Frequency stability of the closed loop frequency tracking system, which decides the sensitivity of the sensor, is determined by the CDR resonator intrinsic losses. It is also affected by the amplifier noise, flicker noise, and electrical thermal noise of components in the circuitry. For the digital phase-lock loop implemented around the programmable lock-in amplifier, quantization error from the ADC and residual frequency modulation of the instrument’s local oscillator also contribute to the frequency noise. However, as shown in these experiments, self-heating dominates the frequency fluctuation in the measurements and so significant gains in accuracy can be achieved through correcting for temperature fluctuations during operation, this being of particular relevance in point-of-care devices. The small temperature sensitivity of the device was accounted for by considering absolute resonant frequencies with their corresponding split, giving a measurement tracking accuracy of 2.3 Hz in these 5.8 MHz devices and representing a factor of 33 improvement in measurement sensitivity.

The signal recovery approach demonstrated in this work is applicable to resonant frequency determination of any piezoelectric resonant sensor although for applications which do not utilise differential measurements, frequency shifts resulting from this approach may need to be accounted for. Ultimately, it is resistor tolerance and circuit noise which limit the achievable limit of Q amplification and hence frequency tracking performance.

## Figures and Tables

**Table 1:** Parameters for CDR and associated circuitry.

Parameter	Average	σ_n-1_
L_m_ (mH)	5.34	0.30
C_m_ (fF)	141.5	8.1
R_m_ (Ω)	383	26
C_p_ (pF)	131.8	2.5

**Table 2:** Performance of CDR with feedback circuitry. An increase in feedback resistance leads to a larger Q, steeper phase transition at resonance and a greater frequency measurement resolution when locking the phase to within 0.05°.

R_f_ (Ω)	I_m_ (µA)	Frequency Shift (Hz)	Q	Phase Gradient (°/Hz)	Frequency Resolution (Hz/0.05°)
No feedback	2.6	0	521	0.0098	5.08
224	6.3	−957	1485	0.0289	1.73
269	8.8	−1035	2283	0.0444	1.13
311	13.9	−1544	4357	0.0860	0.58

**Figure A1:**
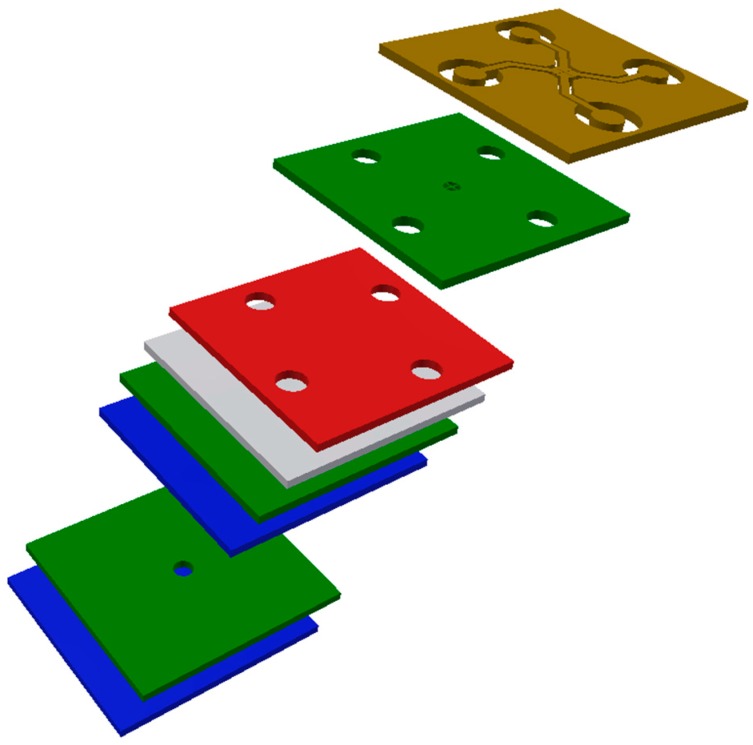
Constituent layers of the piezoelectric CDR biosensor.

## Appendix A

### A.1. Review of Flexural Piezoelectric Biosensor Designs

Flexural resonators aim to measure mass changes due to immobilization of specific biomolecules onto their surface. Changes in surface mass are determined by a measure of the change in resonant frequency of the device. Flexural resonator designs have high quality factors and as they can operate at relatively low frequencies, electronic signal recovery becomes cheaper [39]. A range of flexural piezoelectric biosensor designs have been proposed. Hwang et al. [8] developed a nanomechanical cantilever with PZT actuation. For these 31 kHz resonators, frequency changes down to 94 Hz were recorded for PSA antigen concentrations of 1 ng·mL^−1^. Lee et al. [9] looked to measure C-reactive protein (CRP) with thin film PZT/silicon nitride cantilevers. CRP antibody site ratios down to 10% gave a 117 Hz change in the 28.9 kHz resonant frequency of the devices. Lee et al. [12] investigated a PZT actuated micro cantilever for protein and DNA detection. Mass levels down to 0.46 fg were detected. Shin et al. [13] investigated a multisized PZT based micro cantilever biosensor array. Upon immobilization of IgG molecules, resonant frequency shifts ranging from 44 Hz for the 34 kHz resonators and 19.2 kHz for the 3.3 MHz resonators were measured. Xu et al. [14] developed a PZT/SiN membrane. Measured frequency changes were as low as 150 Hz for these 90 kHz resonators during immobilization of 25 µg/mL goat immunoglobulin G. Gil et al. [10] have fabricated a tuning fork design with an AlN layer for excitation. Devices demonstrated resonant modes up to 800 kHz showing quality factors in excess of 4000 in air. Lu et al. [11] fabricated piezoelectric ZnO micro membranes with mass sensitivities of up to 34 kHz/µg being predicted for resonant frequencies up to 100 kHz. Xu et al. [15] investigated the use of micro-piezoelectric immunoassay chips for simultaneous detection of Hepatitis B virus and α-fetoprotein. For these 70 kHz resonators, frequency shifts of the order 100 Hz were seen for antibody concentrations of 0.1 ng/mL. A move away from flexural mode devices sees researchers targeting direct use of the biosensors within a liquid environment. For example, the 2.36 MHz piezoelectric rotational mode disk resonators by Mehdizadeh et al. [16] demonstrated a 3800 ppm frequency shift after forming monolayers of mercaptohexanol on its surface. A comprehensive review of piezoelectric microelectromechanical resonant sensors for chemical and biological detection is given by Pang et al. [40].

### A.2. Comparison of Piezoelectric and Electrostatic Actuation

Table A1 summarises the advantages and disadvantages of the piezoelectric CDR resonator compared to the capacitively based CDR.

**Table A1:** Comparison of piezoelectric and capacitive CDR sensors.

Parameter	Piezoelectric	Capacitive
Fabrication	Simple design as enclosed cavity under resonator is not a necessity. Moderate number of processing steps. Thin film PZT deposition is a developing technology and there is no current established process for this.	Complicated design and fabrication due to having to produce a sealed cavity under the resonator further complicated by electrical connections needed to both of the bonded wafers. High number of processing steps, but all well established.
Resonator quality factor	Grain boundaries reduce Q factor of resonators.	High crystallinity of the pure silicon structure allows for high Q resonators.
Electro-mechanical coupling factor	High.	Limited due to difficulties in fabricating sufficiently small interelectrode spacing.
Surface roughness ^1^	Poor due to grain size.	Very good as consists of polished silicon and thin metal deposition.
Stability ^2^	Can have poor long term stability due to their porosity [41].	High due to their crystallinity.
Reliability ^2^	Strained layers during fabrication will increase probability of device failure. Actuation arrangement can degrade piezoelectric coefficients rapidly [42].	High due to their crystallinity and thermal expansion coefficient matching during fabrication.
System integration	Good signal to noise ratio allows for simpler electronic detection schemes [this work].	Poor signal to noise ratio results in complex signal recovery electronics [4].

^1^ Biosensing applications typically require a surface roughness of ~1 nm to allow for functionalisation of the surface with self-assembled monolayers; ^2^ As biosensors are a disposable technology, long term reliability is not an issue to consider.

### A.3. Schematic of the Sensor

Figure A1 shows a schematic of the various layers used to fabricate the sensor. Layer thickness is not to scale and is for illustrative purposes only. Layers, from bottom to top, are: (i) silicon substrate; (ii) patterned oxide layer defining cavity region; (iii) continuous silicon layer forming the diaphragm; (iv) continuous oxide layer providing electrical insulation; (v) continuous platinum layer forming ground contact electrode; (vi) patterned PZT layer; (vii) patterned oxide layer; and (viii) patterned gold layer forming top electrode contacts and a top ground plane.

### A.4. Electrical Equivalent Circuit Model

It is widely known that for a piezoelectric thin film employed as actuation and sensing of a resonator with electrodes placed perpendicular to the poled 3-direction, the relationship between the mechanical and electrical variables governed by the constitutive equation of piezoelectric material can be simplified, as shown in [43], as:

(A1) $$\left[\begin{array}{c} S_{1}\\ D_{3} \end{array}\right]=\left[\begin{array}{cc} s_{11}^{E} & d_{31}\\ d_{31} & \varepsilon_{33}^{T} \end{array}\right]\left[\begin{array}{c} T_{1}\\ E_{3} \end{array}\right]$$

Here, *S*_1_ and *D*_3_ are material strain of the diaphragm in the 1-direction and dielectric displacement in the 3-direction respectively caused by stress *T*_1_ applied along 1-direction and electrical field *E*_3_ applied along 3-direction via electrodes. $s_{11}^{E}$, *d*_31_, and $\varepsilon_{33}^{T}$ are elastic, piezoelectric, and dielectric constants of the piezoelectric film. This equation can be rewritten with strain and electrical field as drives with mechanical stress and charge related electrical displacement as outputs:

(A2) $$\left[\begin{array}{c} T_{1}\\ D_{3} \end{array}\right]=\left[\begin{array}{cc} 1/s_{11}^{E} & -d_{31}/s_{11}^{E}\\ d_{31}/s_{11}^{E} & \varepsilon_{33}^{T}-{d_{31}}^{2}/s_{11}^{E} \end{array}\right]\left[\begin{array}{c} S_{1}\\ E_{3} \end{array}\right]$$

The equations of motion for a resonator excited and sensed by piezoelectric thin films can be described by the actuation and sensor equations of the electroelastic system:

(A3) $$M\ddot{x}+\;c\dot{x}+\;Kx=\;-d_{31}/s_{11}^{E}E_{3}$$

(A4) $$d_{31}/s_{11}^{E}\;S_{1}+\;\left(\varepsilon_{33}^{T}-{d_{31}}^{2}/s_{11}^{E}\right)\;E_{3}=D_{3}=\frac{q}{A}$$

Here *M*, *c*, and *K* are the modal quantities of the diaphragm mass, including PZT film and electrode layers, damping, and stiffness of the whole resonating structure respectively. *A* is the area of the electrode. The generated charge *q* is related to the electrical displacement *D*_3_ by the area of the electrode. The voltage generated across the sensor electrodes is related to the charge by the capacitance between the top and bottom electrodes of the sensor.

The resonant system can be represented with a bulk electrical equivalent circuit model, by relating electrical current with mechanical velocity of vibration, $\dot{x}$, by defining the motional current, *I_mot_*, as:

(A5) $$\;I_{mot}=H\dot{x}$$

with *H* the proportionality constant. The motion equation can be written as an RLC electrical model equation:

(A6) $$L_{m}\;{\dot{I}}_{mot}+\;R_{m}I_{mot}+\;\frac{1}{C_{m}}\int^{}I_{mot}dt=u_{ac}$$

(A7) $$I_{mot}+\;C_{p}\frac{du_{ac}}{dt}=I$$

with $L_{m}=M/H^{2},\;R_{m}=r\;/H^{2},\;C_{m}=H^{2}/K$, and u_ac_ the excitation voltage signal.
